# Dysphagia During Pharyngeal and Esophageal Phase of Seronegative Immune-Mediated Necrotizing Myopathy: A Case Report

**DOI:** 10.7759/cureus.81018

**Published:** 2025-03-22

**Authors:** Naoko Takaku, Koji Hayashi, Asuka Suzuki, Yuka Nakaya, Mamiko Sato, Toyoaki Miura, Ichizo Nishino, Yasutaka Kobayashi

**Affiliations:** 1 Department of Rehabilitation Medicine, Fukui General Hospital, Fukui, JPN; 2 Department of Neuromuscular Research, National Institute of Neuroscience, National Center of Neurology and Psychiatry, Tokyo, JPN; 3 Graduate School of Health Science, Fukui Health Science University, Fukui, JPN

**Keywords:** achalasia esophagus, dysphagia', immune-mediated inflammatory myopathy, immune-mediated necrotizing myopathy, videofluoroscopic swallowing studies

## Abstract

Immune-mediated necrotizing myopathy (IMNM) is a category of inflammatory muscle diseases that was identified as distinct from polymyositis. The majority of IMNM cases involve the presence of myositis-specific autoantibodies, specifically anti-signal recognition particle (anti-SRP) or anti-3-hydroxy-3-methylglutaryl-coA reductase (anti-HMGCR). However, some IMNM patients do not exhibit these antibodies, which is referred to as seronegative status. Swallowing difficulties are a common symptom in muscle diseases, including IMNM. However, there is a lack of research specifically investigating the swallowing disorder profile in patients with antibody-negative IMNM.

We describe an 88-year-old Japanese woman case of seronegative IMNM with dysphagia. She developed pain and muscle weakness in the right shoulder followed by muscle weakness in the bilateral shoulder muscles and lower limbs. One year after initial onset, head ptosis and dysphagia developed and she visited our hospital. Based on muscle biopsy and blood tests, she was diagnosed with IMNM. At age 90, she was admitted to our hospital for treatment and received immunotherapy including intravenous methylprednisolone, oral prednisolone, methotrexate and intravenous immunoglobulin therapy. A swallowing screening test immediately after admission revealed coughing during water drinking, but this improved with thickened fluids. The patient continued to experience choking during meals, prompting a videofluoroscopic swallowing study (VF). VF showed decreased clearance of food residue during the pharyngeal phase and retention and reflux during the esophageal phase. Despite immunotherapy, the patient did not experience any significant improvement in clinical symptoms, including limb strength or dysphagia.

This case report highlights dysphagia in a seronegative IMNM patient, demonstrating food retention and reflux in the lower esophagus and decreased pharyngeal clearance on VF. This suggests potential esophageal smooth muscle damage due to an autoimmune mechanism. Further research on dysphagia in IMNM, particularly in the seronegative type, is crucial to understand the pathogenesis and develop effective treatment strategies.

## Introduction

Immune-mediated necrotizing myopathy (IMNM) is a distinct subtype of idiopathic inflammatory myopathies (IIMs), characterized by prominent muscle fiber necrosis and minimal or absent perivascular inflammatory cells [1]. IMNM is differentiated from other IIMs by the presence of specific autoantibodies, including anti-signal recognition particle (SRP) and anti-3-hydroxy-3-methylglutaryl-coenzyme A reductase (HMGCR) antibodies [1,2]. Currently, four pathogenic targets of SRP antibodies are recognized: SRP19, SRP54, SRP72 proteins, and 7SL RNA, while HMGCR antibodies are associated with statin use [2]. Additionally, seronegative IMNM is now recognized as a new subtype [1,2]. All groups exhibit similar characteristics, typically presenting as acute or subacute proximal muscle weakness with a symmetrical pattern, often accompanied by myalgia [2]. Patients with anti-SRP positive IMNM experience more severe muscle weakness than those with anti-HMGCR positive or seronegative IMNM, and are at increased risk for cardiac involvement, interstitial lung disease, and dysphagia [3,4]. Conversely, patients with anti-HMGCR positive IMNM primarily exhibit muscle weakness without extra-muscular involvement [3]. While seronegative IMNM remains less understood, evidence suggests that patients in this category may have an elevated risk of cancer compared to antibody-positive patients [2,5].

Dysphagia is relatively common in IMNM patients, occurring in 20-35% of individuals, including those with the seronegative type [6-8]. While several case reports of IMNM-associated dysphagia exist [7-11], there are no detailed reports on dysphagia in antibody-negative IMNM, including videofluoroscopic swallowing study (VF) profiles. This report describes a case of seronegative IMNM with dysphagia and details its VF findings.

## Case presentation

**Figure 1:**
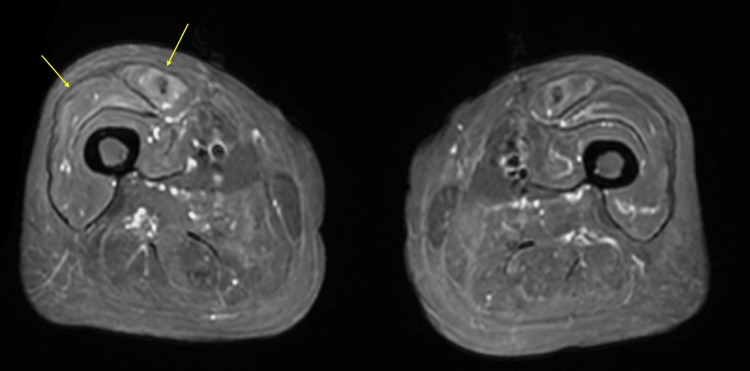
Short τ inversion recovery (STIR) sequence of muscle magnetic resonance imaging (MRI) of the thigh. The muscle MRI reveals hyperintensities in the right rectus femoris and vastus lateralis (arrows), which are consistent with immune-mediated necrotizing myopathy (IMNM).

**Figure 2:**
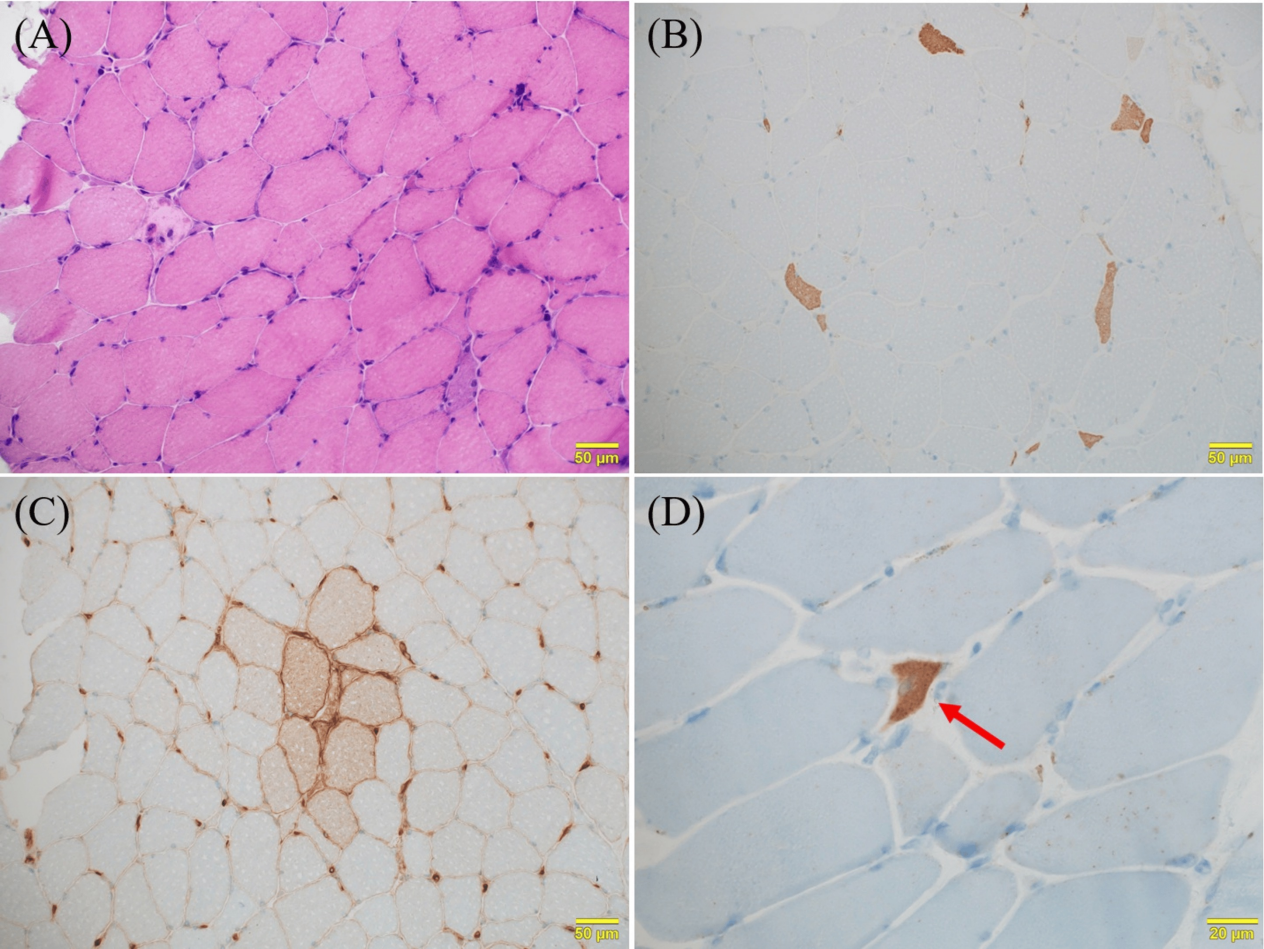
The muscle biopsy findings in the right vastus lateralis and rectus femoris. (A) Hematoxylin and eosin (H&E) staining reveals that muscle fibers exhibit variation in size, with scattered necrotic and regenerating fibers observed. Infiltrating mononuclear cells are not prominently evident. (B) MHC-n staining shows numerous positive cells indicative of regenerating fibers. (C) HLA-ABC staining reveals high expression in some muscle fibers. (D) Immunostaining for p62 highlights fibers exhibiting a granular staining pattern for p62 (arrow).

An 88-year-old Japanese woman with a history of appendicitis, hypertension, and dyslipidemia developed right shoulder pain, followed by muscle weakness in the right shoulder. She had no family history of neuromuscular disorders. She visited a previous hospital, where biceps brachii tears were identified. At the age of 89, she developed muscle weakness in her lower limbs. Although she underwent right shoulder arthroplasty, the improvement in muscle weakness was limited. Over several months, she experienced progressive muscle weakness in the bilateral shoulder muscles and lower limbs. One year after the onset of her initial symptoms, head ptosis and left rotator cuff tears were noted. Additionally, she began to experience choking when consuming fluids. Due to suspected neuromuscular diseases, she visited our hospital. The neurological examination revealed no blepharoptosis, muscle weakness in the face, fasciculations or ataxia; however, the sternocleidomastoid, trapezius, neck flexors, and extensors, as well as the proximal muscles of the upper and lower limbs, were at the Medical Research Council (MRC) grade 2 level, while the distal muscles of the upper and lower limbs were at grade 4-5 level. Sensation and autonomic function were normal. Areflexia was also noted. Blood tests revealed significantly elevated aspartate aminotransferase (AST) of 141 IU/L, alanine aminotransferase (ALT) of 37 IU/L, lactate dehydrogenase (LDH) of 484 IU/L, and creatine phosphokinase (CPK) of 791 U/L. Magnetic resonance imaging (MRI) of the thigh revealed hyperintensities in the muscle parenchyma of the right rectus femoris and vastus lateralis, indicating edema-like changes (Figure 1). Needle electromyography (nEMG) in the right vastus lateralis revealed findings consistent with myopathic changes, including early recruitment during weak contractions, and lacked acute denervation findings such as positive sharp waves or fibrillation potentials. A muscle biopsy was performed at our hospital and assessed at the National Center of Neurology and Psychiatry in Tokyo, Japan. The pathological findings of the muscle biopsy revealed size variations in the muscle fibers, along with scattered necrotic and regenerating fibers; however, infiltrating mononuclear cells were not prominently evident. Positive staining for MHC-n or HLA-ABC was observed in some fibers. Immunostaining for p62 highlighted fibers exhibiting a granular staining pattern for p62 (Figure 2). Both anti-SRP and anti-HMGCR antibodies were negative. Although autoantibodies were negative, the diagnosis of seronegative IMNM was made based on the presence of edema-like changes in the muscle parenchyma observed on MRI, along with necrotic and regenerating changes in muscle fibers and mild expression of HLA-ABC detected in the muscle pathology, as well as the granular staining pattern of p62 in the cytoplasm of muscle fibers reported in IMNM.

At age 90, she was admitted to our hospital for treatment. Upon admission, statins were discontinued, and the patient received intravenous methylprednisolone (1000 mg/day for three days) followed by oral prednisolone (PSL) (1 mg/kg/day). PSL was tapered every two weeks. Methotrexate (MTX) was added on day 22 of admission.

A swallowing screening test immediately after admission revealed coughing during water drinking, but this improved with thickened fluids. The patient continued to experience choking during meals, prompting a videofluoroscopic swallowing study on day 27 after admission. VF showed decreased clearance of food residue during the pharyngeal phase and retention and reflux during the esophageal phase (Video 1). For the prolonged dysphagia, the patient received intravenous immunoglobulin therapy (IVIg; 400 mg/kg/day for five days) on day 28. Despite immunotherapy, the patient did not experience any significant improvement in clinical symptoms, including limb strength or dysphagia. She was discharged from our hospital to an elderly care facility on day 63 after admission.

**Video 1:** Videofluoroscopic Swallowing Study (VF) Findings The VF shows that soft jelly remains on the posterior pharyngeal wall due to decreased clearance during the pharyngeal phase (0:27-0:39). The coronal section VF findings reveal retention and reflux of soft jelly in the lower esophagus, with no esophageal dilation observed (0:41-1:35).

## Discussion

This is the first report documenting the VF findings in seronegative IMNM. The patient presented with dysphagia following shoulder muscle weakness. A swallowing screening test conducted after admission revealed that the patient choked on water, although she was able to successfully swallow thickened fluids. VF findings revealed decreased clearance of food residue during the pharyngeal phase and retention and reflux during the esophageal phase.

Pathologically, IMNM is characterized by scattered necrotic and regenerating muscle fibers [12]. The inflammatory infiltrate primarily consists of macrophages, with only a small number of lymphocytes present [12]. Immunohistochemical analysis reveals mild expression of HLA-ABC, along with the deposition of C5b-9 on the sarcolemma, suggesting that myofiber necrosis is likely driven by antibody-mediated activation of the classical complement pathway [12]. Additionally, the presence of sarcolemmal p62 positivity, characterized by a diffuse pattern of tiny dots, represents a distinctive finding in IMNM [12]. Based on these observations, the pathological features observed in our case were consistent with those previously reported in IMNM.

IMNM treatment aims to reduce muscle inflammation and necrosis and promote muscle regeneration [2]. The primary approach involves corticosteroids (1 mg/kg/day) combined with an immunosuppressant, usually methotrexate (0.3 mg/kg/week) [2]. For severe cases, IVIg or plasmapheresis may be added. Rituximab may be used for anti-SRP-positive IMNM [2]. Other immunosuppressants like azathioprine, mycophenolate mofetil, cyclophosphamide, or cyclosporine may be used for relapses [2]. Treatment success is monitored by clinical muscle strength assessment, muscle MRI, and blood levels of muscle enzymes [2]. In our case, following this treatment plan, corticosteroids and MTX were administered; however, due to limited improvement in swallowing difficulties, IVIg was added to the regimen. However, other immunotherapy options were not considered given the patient's condition and the possibility of discharge to a nursing home.

Dysphagia in IMNM patients may be the first symptom and may be severe, especially if the pharyngeal muscles are affected, leading to difficulty and pain in swallowing and potentially an increased risk of aspiration pneumonia [8,13]. Moreover, since IMNM is an autoimmune-related muscle disease, it can affect not only the muscles involved in swallowing but also the muscles used for phonation, such as the vocal cords [13]. Additionally, edema, stranding, and exudates in the pharynx have been reported in IMNM patients with anti-HMGCR antibodies, contributing to dysphagia [8]. However, we could not find any literature on IMNM cases with VF findings similar to those in our study.

Some reports of IMNM have previously included VF or fiberoptic endoscopic evaluation of swallowing (VE). Khan et al. found that esophagogastroduodenoscopy showed a spastic lower esophageal sphincter, indicating potential achalasia in a patient with PL-7-associated IMNM [7]. The patient received Botox injections, but there was no significant relief. Chaudhry et al. reported two cases of IMNM with anti-HMGCR positivity, one of which described VF findings [9]. In this case, the VF study revealed silent aspiration with liquids, a lack of cough reflex, and mild improvement in swallowing after immunotherapy that included IVIg, steroids, and mycophenolate. Improvement in swallowing function through immunotherapy has also been reported in other cases. Ngo et al. described a case of anti-HMGCR positive IMNM presenting with generalized fatigue, weakness, and dysphagia, which improved with intravenous steroids, plasmapheresis, and IVIg, although the authors did not report VF or VE findings [8].

In our case, the VF findings showed food retention and dilation in the lower esophagus, with no signs indicative of achalasia, including significant esophageal dilation or a bird-beak-like stricture in the lower esophagus. Food retention and reflux in the esophagus may be caused by obstruction of peristalsis due to damage to the esophageal smooth muscle [14]. Although the observation period of our patient may have been short, she did not experience immediate subjective improvement in her dysphagia after immunotherapy. Although it remains unclear whether these findings are specific or not to seronegative IMNM, the VF findings and clinical courses differ from those reported previously. Further studies and the accumulation of reports on IMNM cases with dysphagia, particularly in the seronegative type, are needed to clarify the underlying mechanisms for the development of dysphagia in IMNM cases.

This case has several limitations. The first is the lack of follow-up examinations for VF. Although there was no subjective improvement in dysphagia after immunotherapy, the possibility of improvement based on imaging findings cannot be discounted. It is also important to note that alternative therapies or prolonged immunotherapy were not explored due to the patient's clinical status and transfer to a nursing home. Further VF studies could have provided valuable insights into treatment response and the potential for functional improvement. The second limitation is that long-term follow-up after treatment was not possible, as the patient was admitted to a nursing home, leaving the long-term effects of immunotherapy unknown. The third limitation is the absence of follow-up studies on physiological and imaging findings, including nEMG and muscle MRI, after immunotherapy. If this information had been available, the presentation would have been more compelling.

## Conclusions

In this report, we present a detailed case of dysphagia in a patient with seronegative IMNM, including VF findings. Our case highlights the presence of food retention and reflux in the lower esophagus as well as decreased clearance of food residue during the pharyngeal phase, as demonstrated by VF. This suggests that dysphagia in IMNM may develop through an autoimmune mechanism that leads to damage of the esophageal smooth muscle. Further studies and accumulation of reports on IMNM cases with dysphagia, particularly in the seronegative type, are needed to clarify the underlying mechanisms responsible for dysphagia development in IMNM. This will help to better understand the pathophysiology of dysphagia in this patient population and inform future treatment strategies.
